# Supplementary material for: Comprehensive analysis of the potential effect and mechanism of pyroptosis-related genes in treatment-related myeloid tumors
Source: PLoS One. 2026 Feb 24;21(2):e0343525. doi: 10.1371/journal.pone.0343525 (PMC12931785; doi:10.1371/journal.pone.0343525)
Supplement: Attachment — Submitted filename: Response to Reviewers.docx [file pone.0343525.s008.docx]

Dear Dr. Kota V Ramana and Reviewers,

Thank you for your valuable comments and suggestions on our manuscript. We greatly appreciate the time and effort you have dedicated to reviewing our work, entitled “**Comprehensive analysis of the potential effect and mechanism of pyroptosis-related genes in treatment-related myeloid tumors** (PONE-D-25-50021)”. In response to the reviewers’ comments, we have conducted extensive revisions to the manuscript. Below, we provide a point-by-point response to each comment. We have addressed all concerns raised, and the revisions are highlighted in the revised manuscript. We hope that the revised version meets the journal's standards and is suitable for publication.

**Response to Reviewer #1**

**Major Concerns:**

**1. Critically Small Sample Size: The study is based on only 9 samples (6 t-MN, 3 controls). This severely limits statistical power and increases the risk of false positives. The stringent cutoffs (|logFC| > 3, FDR < 0.05) do not mitigate this fundamental flaw. The authors must acknowledge this as a major limitation and frame all findings as highly exploratory.**

**Response:** We thank the reviewer for this important point. We acknowledge that the sample size is small, which is a limitation of this exploratory study. However, due to the rarity of t-MN, publicly available datasets with high-quality sequencing samples are limited. To mitigate this, we used stringent thresholds (|logFC| > 3, FDR < 0.05) and multiple bioinformatics methods (e.g., GO/KEGG, GSEA, PPI, ROC) to cross-validate the results. In the revised manuscript, we have explicitly stated this limitation in the "Discussion" section and emphasized the exploratory nature of our findings. We have also discussed plans for future validation with larger cohorts. The specific modifications are as follows:

Although this study advances our understanding of the pyroptosis-related mechanisms in t-MNs, several limitations must be acknowledged. The relatively small sample size (n=9) may limit the statistical power and generalizability of our findings, potentially increasing the risk of overfitting and reducing the robustness of the biomarker signatures identified……Given the exploratory nature of this study, our conclusions should be interpreted as hypothesis-generating rather than definitive. Future studies with expanded cohort sizes and experimental validations are warranted to verify the reproducibility and clinical relevance of these findings.

**2. Lack of Validation: The findings are not validated in any independent dataset. To demonstrate potential clinical relevance, the authors must test their hub gene signature in publicly available human t-MN datasets (e.g., GSE14468, GSE6861) and report performance metrics (AUC, sensitivity, specificity).**

**Response:** We fully acknowledge the importance of independent dataset validation for the robustness of the findings. However, the number of publicly available, high-quality human t-MN transcriptomic datasets is extremely limited at present. Furthermore, the sample sources, detection platforms, and sequencing depths of most existing datasets are substantially heterogeneous compared to the data used in this study. Direct integration or comparison may lead to batch effects and biases, thereby compromising the reliability of the analysis. Therefore, this study focuses on conducting a systematic exploration using the available high-quality dataset (GSE135866) and enhances the credibility of the results through cross-validation via multi-level bioinformatics analyses (including GO/KEGG, GSEA, PPI, and ROC). As above comment 1, we have explicitly stated this limitation in the "Discussion" section and discussed plans for future validation with larger cohorts.

**3. Species Extrapolation and Clinical Relevance: All data are from mouse bone marrow. Claims of clinical significance are premature. The discussion must explicitly state that these are preclinical findings requiring validation in human patients.**

**Response:** We thank the reviewer for this insight. We have revised the manuscript to clarify that our findings are based on mouse data and are preclinical. We have added text in the Abstract and Discussion sections to emphasize the exploratory nature and the need for human validation. Clinical relevance is now discussed more cautiously. The specific modifications are as follows:

In “Abstract” section: Collectively, this study delineates the pyroptosis-related molecular landscape of t-MN, uncovering potential biomarkers and therapeutic targets to improve patient outcomes. It is important to note that this study is based on mouse bone marrow data and constitutes a preclinical exploratory analysis. The findings require further validation in human samples and clinical cohorts to enhance their clinical relevance. Clinical validation and exploration of targeted interventions are warranted.

In “Discussion” section: Although this study advances our understanding of the pyroptosis-related mechanisms in t-MNs, **it is essential to emphasize that this research is based on mouse bone marrow data, representing a preclinical exploratory analysis.** Several limitations must be acknowledged.

**4. Questionable Pyroptosis Specificity: The gene list includes pleiotropic regulators (e.g., Trp53, Mtor). The authors must provide evidence linking these genes directly to pyroptosis.**

**Response:** We appreciate this comment. We acknowledge that these genes have broad functions and have toned down claims about pyroptosis specificity, focusing instead on their potential involvement in t-MN pathogenesis. In the revised manuscript, we have added references (e.g., citing PMID: 37164271, 37047282, 37244286) and discussion on the potential roles of these genes in pyroptosis. The following paragraph were inserted after the paragraph discussing the 46 PRDEGs and before the paragraph on GO/KEGG enrichment.

The identification of *Trp53* and *Mtor* as central hub genes underscores the complex, pleiotropic nature of regulatory networks in t-MN pathogenesis, particularly as they relate to pyroptosis. As master regulators of cellular stress responses, their involvement suggests a critical interface between pyroptotic cell death and other fundamental processes like genomic integrity and metabolic signaling. The tumor suppressor p53, encoded by *Trp53*, is a well-known regulator of apoptosis and ferroptosis, but its role in pyroptosis is increasingly recognized. p53 can transcriptionally activate key components of the pyroptotic pathway, such as genes for gasdermin family members (*GSDME*) and NLRP3 inflammasome components, thereby sensitizing cells to pyroptosis upon cytotoxic stress—a common feature of the chemotherapeutic agents that predispose to t-MN development (63-65). Conversely, mutant p53 may acquire gain-of-function properties that suppress pyroptosis, promoting cell survival and clonal expansion. Similarly, mTOR (mechanistic target of rapamycin) serves as a central integrator of nutrient and energy status, cell growth, and autophagy. The AMPK signaling pathway, identified as enriched in our KEGG analysis, is a primary upstream inhibitor of mTOR. AMPK activation and subsequent mTOR inhibition can promote autophagy, which has a dual and context-dependent relationship with pyroptosis (63). While autophagy can dampen pyroptosis by clearing damaged organelles like mitochondria (thus reducing ROS and inflammasome activation), it can also facilitate the presentation of pyroptotic stimuli. Furthermore, mTOR signaling can directly influence the expression and activity of inflammasome components. Therefore, the dysregulation of *Mtor* observed in our t-MN model likely contributes to an altered pyroptotic threshold, potentially enabling the survival of damaged pre-leukemic cells. The interplay between these pleiotropic genes creates a sophisticated regulatory circuit where DNA damage, metabolic stress, and inflammatory cell death converge, potentially offering novel nodes for therapeutic intervention aimed at selectively inducing pyroptosis in t-MN clones.

**5. Overstated Novelty: Genes like Trp53, Mtor, and FoxO3 are already established players in myeloid malignancies. The study fails to convincingly demonstrate what new insight is gained by labeling them as "pyroptosis-related." A comparison with existing biomarkers (e.g., TP53 mutation status) is essential.**

**Response:** We have revised the Discussion to better highlight the novelty of our approach—focusing on pyroptosis-related mechanisms in t-MN—and compared our findings with existing biomarkers (e.g., TP53 mutations). We now emphasize the integrative bioinformatics analysis as a contribution to generating new hypotheses. As below:

Although genes such as Trp53, Mtor, and Foxo3 are well-established players in myeloid malignancies, our study provides a novel perspective by delineating their specific roles in the pyroptosis pathway within t-MNs. Traditionally, Trp53 mutations are recognized as critical biomarkers in t-MNs, associated with genomic instability and poor prognosis (12, 22). However, beyond its canonical functions in apoptosis and DNA repair, emerging evidence indicates that p53 (encoded by Trp53) actively regulates pyroptosis. For instance, p53 suppresses tumor growth by prompting pyroptosis in non-small-cell lung cancer (59). In the context of t-MNs, where DNA damage is a key initiating factor, this p53-mediated pyroptotic pathway may represent a double-edged sword: it could eliminate damaged cells but also contribute to inflammatory microenvironment alterations that favor leukemogenesis. This pyroptosis-focused mechanism distinguishes our findings from conventional Trp53 mutation studies, which primarily emphasize genomic instability, and highlights the potential for targeting p53-dependent pyroptosis as a therapeutic strategy in t-MNs. Similarly, Mtor, a central regulator of cellular metabolism and growth, has been implicated in myeloid neoplasms through its role in promoting cell proliferation. Our enrichment analysis revealed involvement in the AMPK signaling pathway, which negatively regulates mTOR. Recent studies suggest that mTOR inhibition can induce pyroptosis via dysregulation of autophagy and inflammasome activation (60, 61). In t-MNs, chemotherapy-induced metabolic stress may activate AMPK, leading to mTOR suppression and subsequent pyroptosis, which could influence clonal selection and disease progression. This metabolic-pyroptotic axis offers a new dimension to Mtor’s function beyond its known proliferative effects. Furthermore, FoxO3, a transcription factor involved in oxidative stress response, is often associated with apoptosis in leukemia. Our data indicate its connection to pyroptosis through antioxidant activity and inflammatory regulation. FoxO3 can modulate the expression of pyroptosis-related genes like NLRP3 under oxidative stress conditions (62), suggesting a cross-talk between oxidative damage and pyroptotic cell death in t-MNs. Unlike conventional biomarkers that focus on FoxO3's role in cell cycle arrest, our findings emphasize its involvement in inflammatory cell death pathways, providing a unique angle for targeting the redox-pyroptosis interplay in t-MNs. In comparison to existing biomarkers such as Trp53 mutation status, which serves as a static indicator of genomic damage, our pyroptosis-related hub genes dynamic regulation of cell death and inflammation offers a more functional insight into t-MN pathogenesis. While Trp53 mutations are prognostic, they do not fully capture the complex cell death mechanisms altered by therapy. Our approach identifies actionable pathways (e.g., AMPK-mTOR-pyroptosis) that could be exploited for combination therapies, enhancing the specificity of interventions. Thus, labeling these genes as “pyroptosis-related” not only underscores their pleiotropic roles but also reveals novel mechanistic links that may lead to innovative biomarkers and targeted treatments for t-MNs.

**6. Speculative Immune Infiltration: CIBERSORT analysis on 9 bulk RNA-seq samples is highly unreliable. The authors should either remove this analysis or support it with orthogonal validation (e.g., flow cytometry data from the same model).**

**Response:** We acknowledge the reviewer's valid concern regarding the reliability of CIBERSORT analysis performed on our relatively small dataset (n=9). While CIBERSORT has been widely used in bulk RNA-seq studies to infer immune cell composition, its accuracy can be influenced by sample size and technical variability. In our study, the CIBERSORT analysis was intended as an exploratory investigation rather than a definitive quantitative assessment. The primary goal was to identify potential immune trends in t-MNs that could inform future hypothesis-driven research. We have explicitly addressed these limitations in the manuscript and emphasized the need for experimental validation. As stated in our discussion: “Future studies with expanded cohort sizes and experimental validations are warranted to verify the reproducibility and clinical relevance of these findings.”

**7. The current statement ("available upon request") does not comply with PLOS ONE policy.** **All raw data, processed matrices, and analysis code must be deposited in a public repository (e.g., GitHub, Zenodo) with a DOI.**

**Response:** We have updated the Data Availability Statement to deposit all raw data, processed matrices, and analysis code in a public repository, <https://github.com/Sztu00879/Supporting-materials-for-t-AML.git>

**Minor Comments:**

**1. Fig.1: Add exact gene counts at each filtering step.**

**Response:** Thank you to the reviewers for their suggestions. We understand that increasing the number of genes in each screening step in the flowchart may help with result comprehension. However, considering the overall layout and readability of the main diagram, we have clearly listed the relevant gene numbers and screening criteria in the main text and supplementary materials. Therefore, we no longer add specific values in the diagram to keep the illustration concise and clear.

**2. Fig. 7: Include 95% CIs and DeLong test p-values for ROC curves.**

**Response:** We have provided 95% CIs and DeLong test p-values for Fig.7 in Supporting materials S4 Table.

**3. Language: The manuscript requires thorough professional editing for grammar and clarity.**

**Response:** The manuscript has been professionally edited for language clarity. We provided a Tracked Changes Version.

**4. References: Replace preprint citations with peer-reviewed publications where possible.**

**Response:** All preprint citations have been replaced with peer-reviewed references where possible.

**Response to Reviewer #2**

**1. Sample size and statistical power: The analysis uses 6 t-MN and 3 control samples (mouse). This extremely small sample size undermines differential expression calls, enrichment analyses and downstream classifiers. The authors must discuss power limitations explicitly and temper claims. If possible, obtain/analyses additional datasets or aggregate similar public datasets for validation. If no other datasets exist, perform robust statistical measures (e.g., use moderated statistics carefully, provide effect sizes and confidence intervals) and reframe conclusions as exploratory/hypothesis-generating.**

**Response:** Thank you for the valuable comments. We fully understand that the relatively limited sample size (6 t-MN cases vs. 3 controls) may affect statistical power and the robustness of the results. Since t-MN is a rare disease, the number of high-quality transcriptomic samples currently available in public databases is extremely limited. Moreover, significant heterogeneity across different study platforms makes it difficult to directly combine datasets to enlarge the sample size. Given this, the present study is designed as an exploratory analysis using the existing high-quality dataset, aiming to generate new hypotheses rather than to draw confirmatory conclusions. To improve the reliability of the findings, we have employed stringent filtering thresholds (|logFC| > 3, FDR < 0.05) and multiple layers of bioinformatics methods (e.g., GO/KEGG, GSEA, PPI, ROC) for cross-validation. In the revised manuscript, we will explicitly state the limitation of sample size, include additional explanations regarding statistical power and confidence intervals, and emphasize in the Discussion that the conclusions are exploratory in nature and require further confirmation through larger cohorts and experimental validation in the future. As follows:

Although this study advances our understanding of the pyroptosis-related mechanisms in t-MNs, several limitations must be acknowledged. The relatively small sample size (n=9) may limit the statistical power and generalizability of our findings, potentially increasing the risk of overfitting and reducing the robustness of the biomarker signatures identified……Given the exploratory nature of this study, our conclusions should be interpreted as hypothesis-generating rather than definitive. Future studies with expanded cohort sizes and experimental validations are warranted to verify the reproducibility and clinical relevance of these findings.

**2. ROC analyses-risk of overfitting: Reporting AUC > 0.9 with N=9 is almost certainly optimistic and likely overfitted. Recalculate ROC with appropriate cross-validation (leave-one-out or bootstrap) and report 95% confidence intervals. Preferably perform permutation testing to demonstrate that observed AUCs exceed chance. Do not present AUCs without uncertainty estimates and caveats.**

**Response:** Thank you to the reviewers for your valuable comments. We fully understand that in the case of a small sample size (n=9), there may be a risk of overfitting in ROC analysis. As this study aims to conduct exploratory analysis, the ROC results are mainly used to demonstrate the discriminatory trends of candidate genes in the model rather than as confirmatory diagnostic indicators. In the revised draft, we will present the relevant results in a more cautious manner. We also provided explanations of the 95% confidence interval (CIs) and the P-value of the DeLong test for Figure 7 which was presented in detail in the supplementary materials. At the same time, in the discussion section, we will clearly point out the sample size limit and the potential risk of overfitting to ensure that the conclusion presentation is more objective and rigorous. As follows:

Although this study advances our understanding of the pyroptosis-related mechanisms in t-MNs, **it is essential to emphasize that this research is based on mouse bone marrow data, representing a preclinical exploratory analysis.** Several limitations must be acknowledged. The relatively small sample size (n=9) may limit the statistical power and generalizability of our findings, potentially increasing the risk of overfitting and reducing the robustness of the biomarker signatures identified……

**3. Validation: There is no independent validation cohort or wet-lab validation. At minimum, authors should: (a) search for any related datasets (mouse or human t-MN or therapy-related myeloid neoplasms) and attempt in-silico validation of hub gene differential expression; (b) if unavailable, clearly label findings as exploratory and propose concrete experimental validation (qPCR, western blot, functional assays) in Discussion. Strong claims about biomarkers or therapeutic targets must be softened.**

**Response:** Thank you for the valuable comments. We fully understand the importance of independent datasets or experimental validation for the robustness of the results. Currently, there is a lack of high-quality, well-annotated transcriptomic datasets for t-MN (whether from mouse or human) in public databases, and significant heterogeneity across different platforms makes it challenging to conduct reliable integrated validation. For this reason, the present study has not yet included independent validation analyses. In the revised manuscript, we will explicitly note this limitation, emphasize that the findings are exploratory, and add in the Discussion section a plan for future experimental validation (e.g., qPCR, Western blot, and functional studies). As follows:

Given the exploratory nature of this study, our conclusions should be interpreted as hypothesis-generating rather than definitive. Future studies with expanded cohort sizes and experimental validations are warranted to verify the reproducibility and clinical relevance of these findings. Furthermore, therapeutic strategies predicated on pyroptosis induction risk unintended consequences, as primate models reveal suboptimal bone marrow-targeting efficiency (< 5%) and adaptive upregulation of anti-pyroptotic factors (e.g., *BCL2L1*) in 40% of patient-derived xenograft (PDX) models. Moreover, *CYBB* inhibition may exacerbate susceptibility to infection through NADPH oxidase suppression. These constraints underscore the need for multicenter prospective cohort studies, functional validation using t-MN-specific cellular models, and phase 0/1 trials incorporating spatial and dynamic biomarker monitoring to bridge computational insights and clinical implementation.

**Response to Reviewer #3**

**1. The authors use the article " Blood Cancer Discov . 2020 Apr 20;1(1):32–47. doi: 10.1158/2643-3230.BCD-19-0028 Cytotoxic Therapy–Induced Effects on Both Hematopoietic and Marrow Stromal Cells Promotes Therapy-Related Myeloid Neoplasms " as a data source, in which there are 4 types of core samples and 2 sets of RNAseq data, 6 samples each named as MDS or AML. At the same time, the authors do not indicate which samples (from the available options) were used as control samples and which (6 out of 12) were included by them for the analysis. Therefore, it is not possible to evaluate the results obtained.**

**Response:** Thank you for the reviewer's careful review and valuable comments. The dataset used in this study is GSE135866, which originates from research published in Blood Cancer Discovery (2020;1(1):32–47). Based on the annotation information of this dataset and the description in the original publication, we selected 6 treatment-related myeloid neoplasm (t-MN) samples (including MDS/AML models) as the experimental group and 3 bone marrow samples from untreated, control mice as the control group for analysis. This is now explicitly stated to avoid confusion.

**2.****In the original article, mouse model with a 5q deletion leading to the haploinsufficiency of the Egr1 and Apc genes were used to study the effect of the genotype on the development of MDS or AML.**

**Response:** Thank you to the reviewers for your detailed review and comments. We fully understand the genetic background of the mouse model used in the original study, which has 5q deletion and is accompanied by hemizygous deletion of the Egr1 and Apc genes. However, the analysis focus of this study lies in comparing the transcriptional differences between the treatment-induced t-MN group and the untreated control group in the same model. Therefore, all samples share the same genetic background, and the observed differences mainly reflect the influence of treatment-related factors rather than genotype differences. In addition, the GSE135866 dataset has been widely used to study the molecular characteristics of treatment-related myeloid tumors, and its design purpose is also to explore the transcriptional changes induced by chemotherapy/radiotherapy. Therefore, we believe that this model is still applicable to the exploratory analysis of this study.

**3.The Egr1 gene is involved in the regulation of pyroptosis. (See for examples: (1) Egr1 promotes Nlrc4-dependent neuronal pyroptosis through phlda1 in an in-vitro model of intracerebral hemorrhage. Neuroreport. 2024. Jun 5;35(9):590-600. doi: 10.1097/WNR.0000000000002035. Epub 2024 Apr 17. (2) Lysophosphatidic Acid Induces Podocyte Pyroptosis in Diabetic Nephropathy by an Increase of Egr1 Expression via Downregulation of EzH2 International Journal of Molecular Sciences (IJMS). June 2023. 24(12):9968. doi:10.3390/ijms24129968)**

**Response:** Thank you for the reviewer's thorough review and the provided relevant literature. We fully acknowledge that the Egr1 gene has been reported to be associated with the pyroptosis process in certain specific disease models. However, this gene is not a primary focus of the analysis in the GSE135866 dataset. All samples were derived from mice with the same genetic background, and the Egr1 status is consistent between the experimental and control groups. Therefore, it is not expected to introduce systematic bias into the differential analysis of pyroptosis-related genes in this study. Furthermore, existing research on Egr1-mediated pyroptosis is largely focused on neurological and metabolic disease models, and there is a lack of direct evidence for its role in treatment-related myeloid neoplasms. Based on this, we believe the current model remains suitable for the exploratory analysis of pyroptosis-related genes. In the revised manuscript, we will briefly note the background information on Egr1 and its potential, though unconfirmed, influence.

**4. APC deletion also significantly affects the processes of apoptosis.:**

**• Mutated APC creates a different tumor microenvironment: Defective APC leads to genomic instability and can influence the tumor microenvironment (TME), including immune cell infiltration and inflammatory signaling.**

**• Pyroptosis is a form of inflammatory cell death: Pyroptosis is a highly inflammatory type of programmed cell death that is triggered by various signals, including those in the TME.**

**• Pyroptosis can be influenced by the TME: Studies have shown that pyroptosis scores are correlated with immune cell infiltration and inflammatory markers within the TME, which is shaped by the presence of mutated genes like APC.**

**Thus, the study of the potential effect and mechanism of action of pyroptosis-related genes on such a model is incorrect.**

**Response:** Thank you for the reviewer’s careful review and constructive comments. We understand that deletion of the Apc gene may influence apoptosis and the tumor microenvironment, thereby potentially altering inflammation-related signaling. However, the analysis in this study is based on mouse samples with the same genetic background, and the Apc status is consistent between the experimental and control groups. Therefore, the observed transcriptional differences mainly reflect the effects of treatment-induced factors rather than genotype variations. Additionally, the GSE135866 dataset was originally designed to investigate transcriptomic changes induced by chemotherapy/radiotherapy, and it remains valuable for reference in pyroptosis pathway analysis. In the revised manuscript, we will further clarify the model background and its limitations to ensure the scientific validity and rationality of the interpretation of the results.

**General Revisions**

In addition to point-by-point responses, we have made the following general revisions to comply with PLOS ONE policies:

(1) In ‘Contributors’ section，we have explained the contributions of all the authors.

(2) Revised the Data Availability Statement to provide a non-author institutional contact point and full citation for data access, <https://github.com/Sztu00879/Supporting-materials-for-t-AML.git>

(3) Ensured all code is shared via a public repository: <https://github.com/Sztu00879/Supporting-materials-for-t-AML.git>

We believe that the revisions have significantly improved the manuscript. Thank you again for your constructive feedback. We hope that the revised version is now acceptable for publication in PLOS ONE.

Sincerely,

Xiaohui Zhu
