# Supplementary material for: Comprehensive analysis of the potential effect and mechanism of pyroptosis-related genes in treatment-related myeloid tumors
Source: PLoS One. 2026 Feb 24;21(2):e0343525. doi: 10.1371/journal.pone.0343525 (PMC12931785; doi:10.1371/journal.pone.0343525)
Supplement: Attachment — Submitted filename: Response Letter.docx [file pone.0343525.s009.docx]

Feb. 2, 2026

Dear Reviewers,

Thank you very much for your editorial decision and the reviewers’ thoughtful comments on our manuscript entitled “Comprehensive analysis of the potential effect and mechanism of pyroptosis-related genes in treatment-related myeloid tumors” (PONE-D-25-50021R1). We sincerely appreciate the time and effort invested by you and the reviewers in evaluating our work.

We have carefully considered all comments and made extensive revisions to address the concerns raised—particularly those from Reviewer #3. Below we provide a point-by-point response to each substantive comment. All changes in the revised manuscript are highlighted in the “Tracked Changes Version” file for ease of review.

**Response to Reviewer #1**

**Comment 1:** We have carefully reviewed the revised manuscript entitled "Comprehensive analysis of the potential effect and mechanism of pyroptosis-related genes in treatment-related myeloid tumors" (Manuscript ID: PONE-D-25-50021R1) and the authors’ point-by-point responses to the reviewers’ comments.
The authors have done an excellent job addressing all the concerns raised during the initial review. Their revisions significantly enhance the clarity, methodological transparency, and biological interpretation of the study. Specifically, they have provided additional validation for their bioinformatic analyses, clarified the limitations of their dataset, and improved the discussion regarding the functional implications of the identified pyroptosis-related genes in therapy-related myeloid neoplasms.

All requested clarifications—ranging from statistical methodology to justification of gene selection and cohort characteristics—have been thoroughly and thoughtfully addressed. The revised figures and tables are now more informative, and the supplementary materials adequately support the main conclusions.

I commend the authors for their responsiveness and scientific rigor. In my view, the manuscript now meets the standards for publication in PLOS ONE. No further revisions are required.
Well done!

**Response 1:**

Thank you very much for your careful review of our revised manuscript and for the highly positive and encouraging evaluation.

We sincerely appreciate your acknowledgment of the improvements in this study regarding methodological transparency, interpretation of results, and overall quality, as well as your recognition of our thorough responses to all reviewers' comments.

Your valuable suggestions have played a significant role in refining this research. We are deeply honored and truly grateful to receive your confirmation that the manuscript now meets the publication standards of PLOS ONE.

Once again, thank you for your time and expert guidance.

### Response to Reviewer #3

**Comment 1:** The model used and the number of objects in groups (3+3) do not allow the authors to draw statistically sound conclusions. The comments received cannot change the situation.

**Response 1:** We fully agree that the small sample size (n=3 per group) limits the statistical power and generalizability of our findings. As noted in our revision, this study is an exploratory bioinformatic analysis based on the publicly available GSE135866 dataset. Our goal is not to establish definitive conclusions but to generate testable hypotheses regarding pyroptosis-related mechanisms in therapy-related myeloid neoplasms (t-MNs). We have now explicitly emphasized the exploratory nature of this work in the Abstract, Results, Discussion, and Conclusion sections, clarifying that findings should be interpreted as hypothesis-generating and require validation in larger cohorts or experimental models (see Tracked Changes Version).

**Comment 2:** In the reviewer response authors state that control BM cells, treated and nontreated EA-Trp53 LSKs are all genetically the same. Probably there was some mistake, as in the dataset original article the control set is clearly stated as obtained from wild type C57BL/6 mice, while EA-Trp53 LSKs cells are from engineered Egr1+/−, Apcdel/+ mice, also these cells were additionally transduced with trp53 shRNA bearing lentivirus vector. In other words, “six t-MN samples” should have lower Egr1 and Apc expression and no expression for p53 compared to control set, as it is intended by the group who made the dataset.

Authors of the dataset article state that “To decipher the effects of EA-Trp53 loss, we compared WT control (n = 3) with EA-Trp53 LSK+ samples [includes both the no-ENU and ENU-treated groups; (n = 6); Fig. 1E]. Gene set enrichment analysis (GSEA; ref. 31) of the top enriched curated pathways revealed that cell-intrinsic loss of Egr1, Apc, and Trp53 downregulates oxidative phosphorylation, DNA repair, apoptosis, and cell-cycle checkpoints. Similar results were observed when we compared only the three ENU-exposed EA-Trp53 LSK+ samples with WT controls (Supplementary Fig. S4)”. We see, that Egr1, Apc and p53 expression alterations have substantial transcriptomic influence independent from ENU treatment.

**Response 2:** Thank you for your thorough review and valuable comments.

We acknowledge the oversight in our initial description. We confirm that in the GSE135866 dataset, the control samples are derived from wild-type C57BL/6 mice, whereas the EA-Trp53 LSK cells originate from Egr1^+/−^, Apc^del/+^ genetically engineered mice and are further transduced with a lentivirus carrying Trp53 shRNA. Our previous description of the two sample types as "genetically identical" was not sufficiently precise and may have led to misunderstanding. The analysis in this study is based on the established model design of the original dataset, treating EA-Trp53 LSK cells overall as an experimental model for therapy-related myeloid neoplasms to characterize their transcriptional alterations, rather than comparing effects between different genetic backgrounds.

The functional loss of Egr1, Apc, and Trp53 in EA‑Trp53 LSK samples can itself exert a significant and broad impact on the transcriptome, as clearly described in the original dataset study (PMID: 32924016). This study does not regard ENU treatment as the sole or primary driver of transcriptional changes. Instead, based on the overall comparison between WT controls and EA‑Trp53 LSK cells, we analyze the molecular features associated with pyroptosis and their potential regulatory networks within the context of a therapy‑related myeloid neoplasm model. In other words, our analytical framework focuses on the t‑MN phenotype represented by this composite genetic loss state, rather than attempting to differentiate the effects under ENU versus non‑ENU conditions.

To avoid ambiguity, we provided a more accurate and clear description of sample origin and genetic composition in the revised manuscript, ensuring that the presentation of the study design remains consistent with the original dataset (see Tracked Changes Version).

**Comment 3:** Overall, huge analysis was done and thoroughly described, maybe the article could be made into a bioinformatics pipeline article for in-depth RNA-seq analysis with GSE135866 dataset as example.

**Response3:** While we appreciate this suggestion, our primary aim remains biological insight into pyroptosis in t-MNs, not methodological innovation. We have therefore strengthened the biological narrative throughout the manuscript and clarified that the bioinformatic tools serve as means to address our central research question.

We believe these revisions have substantially improved the rigor, clarity, and contextual framing of our study. Thank you again for the opportunity to enhance our manuscript. We hope the revised version meets the journal’s standards for publication in PLOS ONE.

Sincerely,

Xiaohui Zhu
